# Is meningomyelocele an absolute contraindication for epidural labor analgesia?

**DOI:** 10.1186/s40981-023-00666-0

**Published:** 2023-11-04

**Authors:** Masahiko Bougaki, Kanji Uchida

**Affiliations:** Department of Anesthesiology and Pain Relief Center, the University of Tokyo Hospital, 7-3-1 Hongo, Bunkyo-Ku, Tokyo, 113-8655 Japan

To the Editor,

Meningomyelocele, a type of spina bifida , is widely recognized as a relative contraindication for neuraxial anesthesia. Many anesthesiologists are unwilling to provide epidural anesthesia to patients with a history of meningomyelocele repair. However, it remains uncertain whether such patients must forsake lumbar epidural labor analgesia in case of pregnancy.

A 30-year-old primipara pregnant woman presented to our hospital hoping for epidural labor analgesia after being denied by two other major perinatal centers. She had a history of sacral meningomyelocele that had been repaired at the age of 6 months. Although she had used clean intermittent catheterization since childhood for neurogenic bladder, she was able to lead a normal daily life and was working as a nurse. Her neurological status was unremarkable except for a slight reduction of sensation in the anal and buttock areas. A lumbosacral magnetic resonance imaging (MRI) revealed the following: (1) The spinal cord did not end in a conus medullaris but was elongated and tethered to the sacral subdermal lipoma, and (2) normal epidural space was preserved in the lumbar region (Fig. 1). We assessed that epidural anesthesia could be safely performed; however, spinal anesthesia would need to be avoided to prevent spinal cord injury. At present, our hospital offers only daytime epidural labor analgesia. After discussion with the patient and her husband, she was scheduled for planned induction of labor with epidural analgesia at 38 weeks of pregnancy. We also fully discussed the anesthesia plans for possible cesarean section and confirmed her desire to try epidural anesthesia if the degree of urgency permitted, not general anesthesia from the beginning, in case emergency cesarean section was required.

**Fig. 1:**
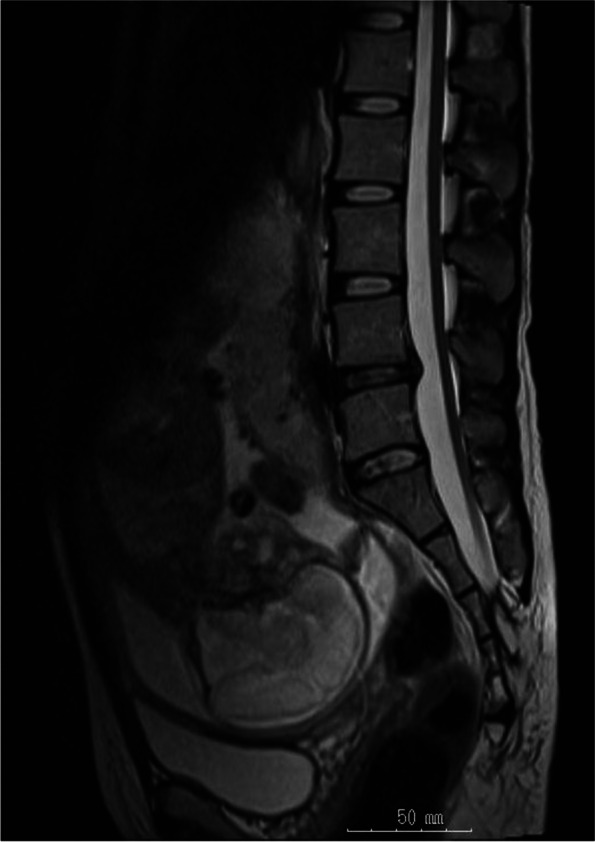
MRI scan of the lumbosacral region. The spinal cord is elongated and tethered to the sacral subdermal lipoma. Normal epidural space is preserved in the lumbar region

Ultimately, epidural labor analgesia was not provided because the patient’s labor spontaneously started during midnight and progressed very smoothly, and she delivered the baby by the morning. However, she was satisfied with our management plan for offering epidural labor analgesia and expressed a desire to use it for her next childbirth.

Several cases of successful use of epidural labor analgesia in parturients with repaired meningomyelocele have been reported [1–4]. However, difficulty of performing an epidural can vary depending on the extent of the spinal lesion and the associated corrective surgery [2, 4], potentially increasing the risk of an inadvertent dural puncture. Furthermore, there might be an increased possibility of poor sacral spread of local anesthetics or an asymmetrical block [2].

An increasing number of parturients are requesting epidural labor analgesia in Japan. Those with a history of meningomyelocele repair should not be routinely denied epidural labor analgesia; however, the feasibility of the procedure should be determined on a case-by-case basis with the aid of MRI data.

## Data Availability

Not applicable.

## Abbreviation

MRI: Magnetic resonance imaging
